# Engineering the Phase Front of Light with Phase-Change Material Based Planar lenses

**DOI:** 10.1038/srep08660

**Published:** 2015-03-02

**Authors:** Yiguo Chen, Xiong Li, Yannick Sonnefraud, Antonio I. Fernández-Domínguez, Xiangang Luo, Minghui Hong, Stefan A. Maier

**Affiliations:** 1 The Blackett Laboratory, Imperial College London, London SW7 2AZ, United Kingdom; 2 Department of Electrical and Computer Engineering, National University of Singapore, 117576, Singapore; 3 State Key Laboratory of Optical Technologies on Nano-Fabrication and Micro-Engineering, Institute of Optics and Electronics, Chinese Academy of Science, Chengdu, 610209, China; 4 Inst. NEEL, CNRS, 25 rue des Martyrs BP 166, 38042 Grenoble cedex 9, France; 5 Departamento de Fisica Teorica de la Materia Condensada and Condensed Matter Physics Center (IFIMAC), Universidad Autonoma de Madrid, E-28049 Madrid, Spain

## Abstract

A novel hybrid planar lens is proposed to engineer the far-field focusing patterns. It consists of an array of slits which are filled with phase-change material Ge_2_Sb_2_Te_5_ (GST). By varying the crystallization level of GST from 0% to 90%, the Fabry-Pérot resonance supported inside each slit can be spectrally shifted across the working wavelength at 1.55 *µ*m, which results in a transmitted electromagnetic phase modulation as large as 0.56*π*. Based on this geometrically fixed platform, different phase fronts can be constructed spatially on the lens plane by assigning the designed GST crystallization levels to the corresponding slits, achieving various far-field focusing patterns. The present work offers a promising route to realize tunable nanophotonic components, which can be used in optical circuits and imaging applications.

The increasing interest in developing ultra-compact optical systems has recently led to enormous research attention on nanophotonic schemes, which are able to manipulate light at the nanoscale. Particularly, metallic nanostructures allow for a deep sub-wavelength electromagnetic confinement which is not possible with conventional dielectric optics^1^,^2^,^3^,^4^,^5^. The planar lens, thanks to its small thickness and excellent focusing capability, has been developed to replace its dielectric counterpart as a paradigmatic nanophotonic component^5^,^6^,^7^,^8^. Several types of planar lenses have been studied so far, for example zone plates^9^,^10^,^11^, nano-slit and nano-hole arrays^5^,^6^,^7^,^8^,^12^,^13^,^14^, photonics crystals^15^ and metasurfaces^16^. Although different terminologies are used in the aforementioned techniques, they share the same principle of focusing, which is to generate a constructive interference at the focal point by curving the phase front of an incident plane wave.

The performance of planar lenses has been optimized through sophisticated designs, however, most of the proposals so far lack the tunability of the focal position once being fabricated. The control of the focal position is important in active optical circuits and miniaturized integrated photonic components, where light-path switching and fine tuning of the optical response are essential for high performance. Various strategies have emerged to enable the tunability of planar lens, in which one wants to manipulate the transmitted phase front from a predefined structure. For instance, varying the angle of the incident light was demonstrated to tune the focal position of a plasmonic lens^17^. Other strategies investigated include the use of anisotropic liquid crystals, or the modulation of the spatial phase of the incident field^5^,^18^. All the above approaches provide the ability to change the focal position or the hotspot within nano-metric length scales, but they can only be implemented through complex controlling systems or sophisticated manipulation of the incident light beams.

Recently, Ge_2_Sb_2_Te_5_ (GST), a phase-change material widely used in commercial optical disks, has been proposed in various tunable plasmonic designs^19^,^20^,^21^. These designs exploit the large refractive index contrast between its amorphous and crystalline phases in the visible and infrared (IR) regions (for example at 1.55 *µ*m, *n* = 4.4 and 7.2 for amorphous and crystalline phases)^22^,^23^,^24^,^25^. GST is non-volatile at room temperature^20^,^21^,^27^,^29^, and can be switched between its two phases within a short time (< 30 ns) by an external stimulus, such as optical pumping or electric current for more than 10^5^ cycles^25^,^26^,^28^. In this article, we propose a novel approach to engineer the phase front at 1.55 *µ*m in the conventional telecom band. By introducing GST into a metallic slit array as shown by the schematic drawing in Figure 1a, we are able to achieve different focusing patterns from a geometrically fixed planar lens. First, a single slit filled with GST in a gold film is theoretically studied, demonstrating an electromagnetic phase modulation larger than *π*/2 via changing the crystallization of GST. By constructing twenty one such slits into a planar lens, the phase front of a plane wave can be further shaped with various curvatures, and we numerically prove both the lateral and longitudinal manipulation of the focus. Finally, a proof-of-concept experimental realization is presented. This planar lens platform provides a promising route to realize tunable micro-/nanophotonic devices.

## Results and Discussion

**Figure 1:**
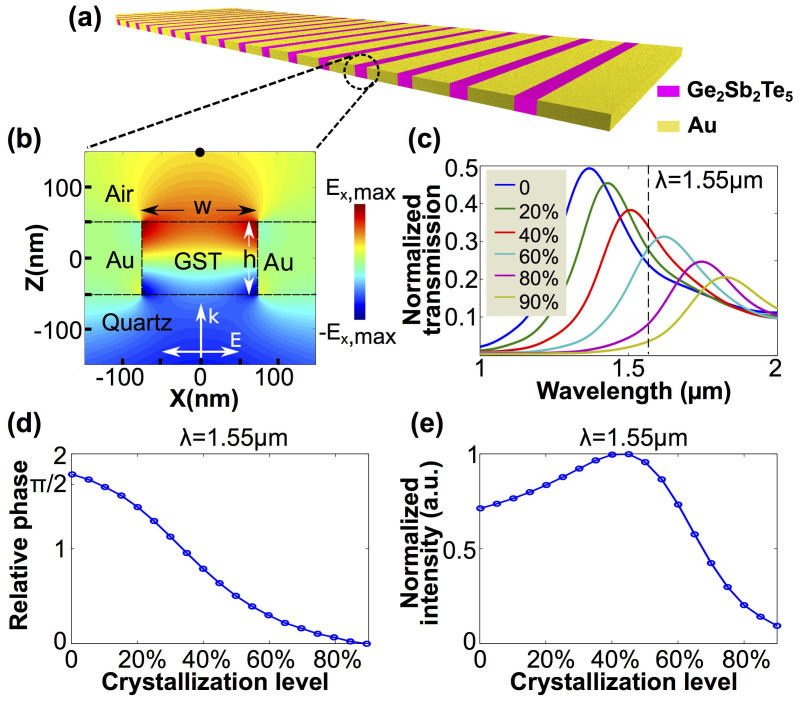
(a) Schematic diagram of the planar lens. (b) The cross-section showing the x-component of the electric field of the 1st order Fabry-Pérot mode from a single slit. The thickness of the Au film *h* is 100 nm, and the width of the slit *w* is 150 nm. A point monitor marked by the black dot is placed on the central axis of the slit and 100 nm above the GST-air interface to record the phases and electric field intensities in panel (d) and (e). (c) The evolution of the single-slit transmission spectrum for different crystallization levels. The spectra are normalized to the transmission from the same slit but filled with air. By varying the crystallization level of the GST, the extraordinary transmission peak of the 1st order Fabry-Pérot mode is shifted across the working wavelength at 1.55 *µ*m. The percentages shown in the legend correspond to the GST crystallization levels. (d) The relative phase and (e) the normalized electric field intensity at 1.55 *µ*m as functions of the crystallization level with a 5% step increment. Both (d) and (e) are evaluated at the point monitor indicated by the black dot in panel (b).

The basic unit of our proposed planar lens is shown by the schematic cross-section in Figure 1b, which is a single slit in a metallic film filled with GST. The structure is supported by a quartz substrate. Transverse magnetic (TM) light excites the structure from the quartz side and radiates into free space on the top. Since the refractive index of GST is higher than the quartz substrate and air superstrate (*n* > 4 for both amorphous and crystalline GST at wavelengths between 1 *µ*m and 2 *µ*m)^30^, the GST-quartz and GST-air interfaces work as mirrors, forming a standing wave cavity which supports a strong Fabry-Pérot (FP) resonance. The colors in Figure 1b render the amplitude of the *x*-component of the electric field, illustrating the 1st order FP mode supported by the structure. The FP mode results in an extraordinary optical transmission (EOT) peak that can be identified in the transmission spectrum^31^,^32^,^33^. The resonant wavelength *λ_FP_* in free space can be roughly related to the refractive index *n* in the slit by *λ_FP_* ∝ (*h*/*N*) × *n* (assuming perfect reflection at the top and bottom ends of the slit), where *h* is the height of metallic film and *N* denotes the order of the FP mode. This expression suggests that this mode can be shifted linearly with *n*.

The refractive index *n* of GST can be tuned to any value between 4.4 (amorphous phase) and 7.2 (crystalline phase) due to the nucleation-dominated nature. Small crystalline nuclei are formed first as GST is heated up above its crystallization temperature (~150°C). These numerous randomly-distributed small crystals will then grow and merge together to form a crystalline structure^34^,^35^. Therefore, GST at the intermediate crystallized levels can be regarded as a mixture of amorphous and crystalline molecules with different proportions. The effective permittivity 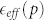 of the partially crystallized GST can be approximated by an effective-medium theory using the Lorentz-Lorenz relation^20^,^35^,^36^, which is defined as 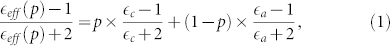
where *p*, ranging from 0 (amorphous) to 1 (crystalline), is the crystallization level, and 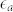 and 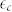 are the permittivities for amorphous and crystalline GST, respectively. The complex refractive indices of GST extracted from Ref. 30 are related to permittivities by 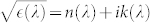.

A detailed numerical analysis of the single-slit optical properties as the crystallization level of GST varies is performed using Lumerical FDTD Solutions. The 2D system is enclosed in perfectly matched layers. A TM polarized plane wave is normally incident onto the structure. A mesh size of 2 nm by 2 nm is used within the slit region. In our design, the film thickness and slit width are *h* = 100 nm and *w* = 150 nm. Figure 1c shows the evolution of the transmission spectrum as GST crystallization level increases in the single-slit geometry as depicted in Figure 1b. These spectra are normalized to the transmission spectrum of a slit with the same geometry but filled with air. The maximum crystallization level considered in the simulation is 90% based on the previous experimental results^20^. Significant spectral shifts of the EOT peak can be observed, as it crosses the telecom wavelength at 1.55 *µ*m. Based on the fact that a phase difference as large as *π*/2 can be achieved by detuning the driving frequency from one side of a resonance to the other side, this spectral shift of the EOT peak suggests that under a monochromatic illumination at 1.55 *µ*m, a considerable phase modulation could be achieved in the transmission field as the crystallization level changes from 0% to 90%.

To confirm this assumption, a point monitor located along the central axis of the slit and 100 nm above the GST-air interface is used to probe the phase and intensity of the transmitted electromagnetic field at 1.55 *µ*m. The relative phase as a function of the GST crystallization level with a 5% step increment is plotted in Figure 1d. Note that we use the result at 90% GST crystallization as reference so that the relative phases are all positive in sign. Due to the resonant spectral shift across the telecom regime, the phase difference around 1.55 *µ*m is found to be 0.56 *π* between 0% and 90% crystalline GST. In contrast, FDTD simulations indicate that a 100 nm thick GST film without any metallic structures can only achieve a phase difference of 0.15 *π* between these two crystalline phases. As a result of the resonant nature, the transmission intensities are non-uniform at different crystallization levels. The normalized intensity of the transmitted field as a function of crystallization level is plotted in Figure 1e, where the maximum value at 45% GST crystallization is used as reference.

The proposed planar lens structure consists of an array of 21 slits like the one discussed above. This periodic array spans *W* = 10 *µ*m laterally and has a center-to-center distance of 500 nm between neighboring slits. Similar to conventional optical lenses, the focusing pattern of a far-field planar lens is governed by the transmitted electromagnetic phase front. The electromagnetic phase distribution at the lens plane (*x*-direction) required to achieve a focus with a nominal focal length *f_nominal_* and a nominal lateral offset *A_nominal_* relative to the lens axis at *x* = 0, can be predicted using the equal optical length principle^6^,^8^

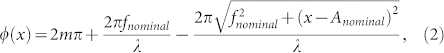
where *λ* is the operating wavelength and *m* is an integer (see Supplementary Information section 2 for more details). To modify the focus, which translates into setting different values to *f_nominal_* and *A_nominal_*, the lens is required to generate distinct transmitted phase fronts based on Eq. 2.

In order to demonstrate that different focusing patterns can be realized by this geometrically fixed planar lens, several examples of varied nominal focal positions are chosen for illustration. First, focuses at different positions along the lens axis are investigated, where *f_nominal_* is changed while *A_nominal_* = 0. Then, the shifting of the focus parallel to the lens plane will be discussed, in which *f_nominal_* is fixed while different *A_nominal_* values are tested. For all the examples demonstrated in this section, a theoretical phase front *ϕ*(*x*) is calculated by Eq. 2 for a given pair of *f_nominal_* and *A_nominal_*. We then choose the GST crystallization level required for each slit with the help of Figure 1d, taking the closest possible value to fit the phase distribution. The source and boundary conditions used in the FDTD simulations for the 21-slit lenses are the same as for the single slit configuration described above. Note that although the electromagnetic phase has a period of 2*π*, only the phases between 0 and 0.56*π* can be achieved in our design. How we assign GST crystallization levels to those slits with *ϕ*(*x*) > 0.56*π* will be discussed later.

**Figure 2:**
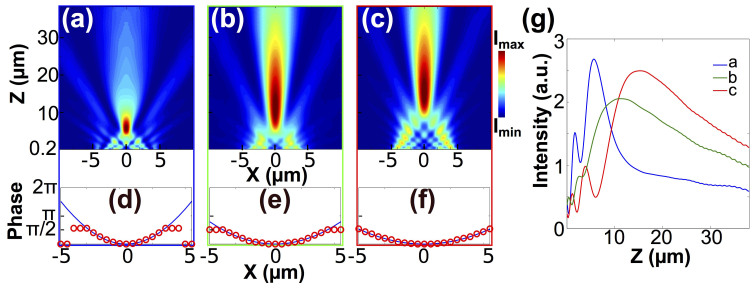
Panels (a), (b) and (c) show the simulated cross-sections of the magnetic field intensity distributions of the planar lens with nominal focal lengths of (a) 10 *µ*m, (b) 20 *µ*m and (c) 30 *µ*m. Panels (d), (e) and (f) show the calculated phase fronts *ϕ*(*x*) (blue curves) and the discrete phase distributions from the single-slit configuration at varied GST crystallization levels (red circles). (g) Magnetic field intensity distributions as a function of the distance from the planar lens surface along the lens axis.

Figure 2a–c illustrate three focusing patterns of magnetic field intensity, in which *f_nominal_* = 10, 20 and 30 *µ*m are used to calculate the phase fronts *ϕ*(*x*) in Eq. 2. A clear trend of the focus shifting away from the lens can be seen as *f_nominal_* increases, accompanied by an elongated depth of focus. In Figure 2d–f, the phase fronts obtained from Eq. 2 are shown in the solid blue curves, and the red circles depict the discrete phase distributions that we use to assign the degree of crystallization (refractive index) to each slit in the array based on Figure 1d. The 1D magnetic intensities along the lens axis of the three cases are shown in Figure 2g. The maximum intensities from the simulation are found to be at 5.8, 11.2, and 15.6 *µ*m away from the lens plane, respectively. The deviation between nominal and simulated focal lengths was expected, and can be attributed to the small Fresnel number (FN) defined as *W*^2^/(*λf_nominal_*) associated with this design^37^.

Let us stress that the theoretical phase front *ϕ*(*x*) from Eq. 2 ranges from 0 to 2*π*, while the maximum phase modulation value achievable from this GST-slit configuration is 0.56*π*. In the following, we discuss briefly how we assign a crystallization level to the slits located at positions *x* at which the nominal *ϕ*(*x*) is out of the feasible range. For 0.56*π* < *ϕ*(*x*) < *π*, 0% crystallized GST, which corresponds to the maximum phase modulation 0.56*π* is used in the slits. On the other hand, for *ϕ*(*x*) ≥ *π*, 90% crystallized GST that corresponds to the minimum phase modulation is used. The reason behind this choice is that when the calculated *ϕ*(*x*) is slightly larger than the maximum achievable value, 0.56*π* still works as an approximation to construct the phase front obtained from Eq. 2. However, for larger *ϕ*(*x*) (≥ *π*, for example), none of the GST crystallization levels would provide a valid phase reproduction. To reduce the impairment on the phase front caused by the phase mismatch, we opt for minimizing the transmitted intensity from these slits. Therefore, GST with 90% crystallization, which has the smallest value in transmission is chosen (see Figure 1e). The cut-off value *π* as discussed above seems to be valid in the configurations considered here, but can be further optimized from case to case.

**Figure 3:**
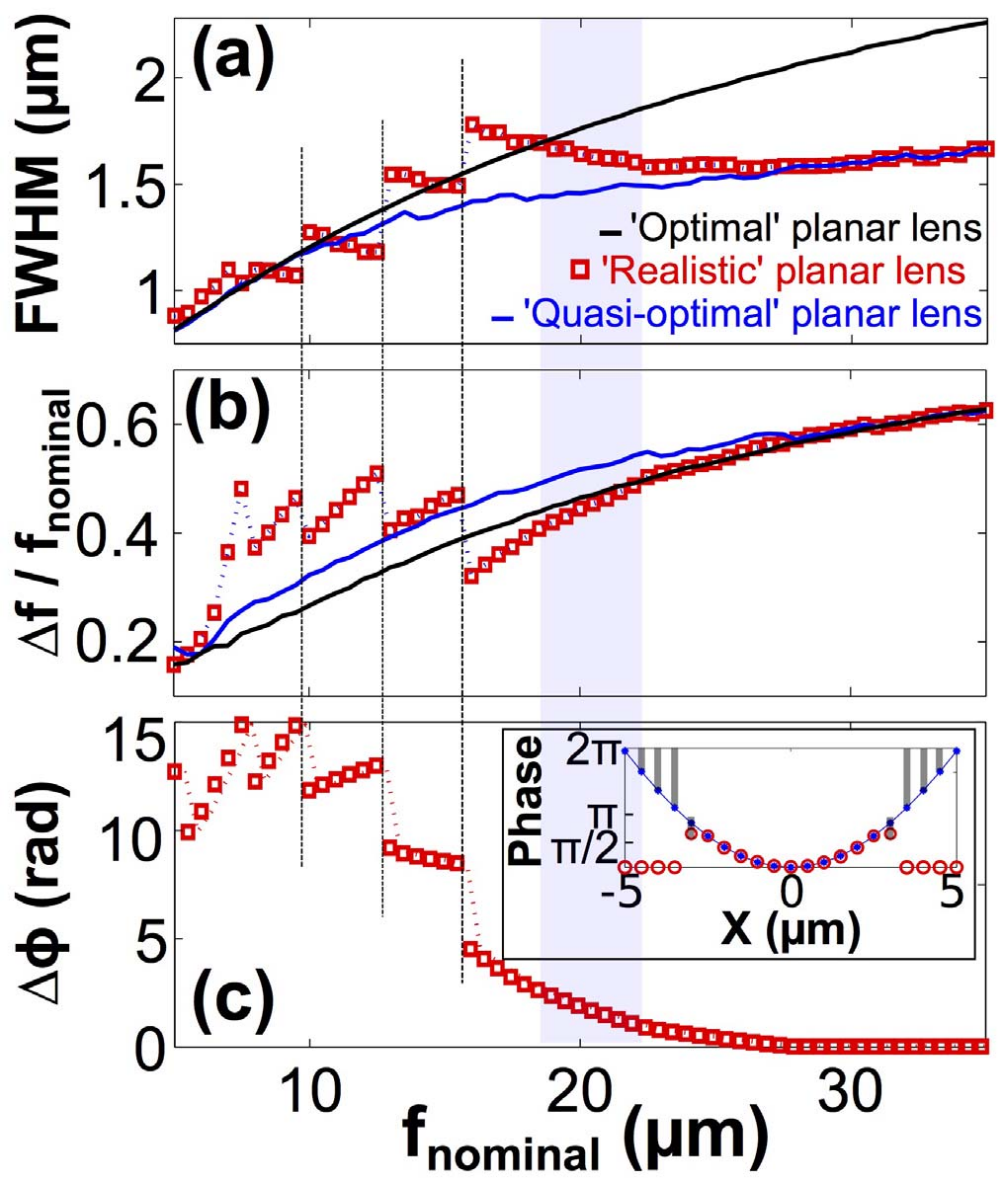
Results from an analytic model showing the performance of the planar lens as functions of nominal focal length *f_nominal_*. Three cases are considered. The red squares denote the ‘realistic' planar lens case that the point sources are assigned with phase and intensity values only from the GST-slit configuration as shown in Figure 1d and e. Blue curves represent the ‘quasi-optimal' case which combines the data based on GST-slit configuration when the required *ϕ*(*x*) is within the phase coverage (*ϕ*(*x*) < 0.56*π*), and sources with phase equal to *ϕ*(*x*) when *ϕ*(*x*) is outside the phase coverage. The ‘optimal' case is plotted in black curves, in which all the sources can radiate at any required *ϕ*(*x*), and they have the same intensity. Panel (a) shows the full-width at half-maximum in cross sections of the focuses along x-axis. Panel (b) illustrates the relative difference between the *f_nominal_* and *f_analytic_* observed in the analytic model. Panel (c) reports the summation of the phase errors from the 21 sources compared with nominal *ϕ*(*x*) in the case of the ‘realistic' planar lens.

To offer more insight into the focusing ability of the planar lens and the described algorithm of assigning GST crystallization levels, a 2D analytic model is constructed for further studies. The 21 slits are represented by 21 point sources, which radiate p-polarized electromagnetic waves at predefined intensities and phases. For simplicity, the angular intensity distribution of the radiation from each source is modeled through Fraunhofer diffraction (the effect of surface plasmons excited on the Au film is not included). The electromagnetic field at any position in the 2D space is a superposition of the waves from the 21 sources. We term ‘realistic' planar lens to the theoretical configuration in which the intensity and phase of each source is determined by the same procedure as in the FDTD simulation (we set the phase values and the corresponding intensities based on Figure 1d and e for the point sources). This ‘realistic' planar lens is compared with two references. The ‘quasi-optimal' planar lens is an improved version of the ‘realistic' planar lens. The source setting is identical to ‘realistic' planar lens if 0 ≤ *ϕ*(*x*) ≤ 0.56*π*. If *ϕ*(*x*) > 0.56*π*, a source with phase equal to *ϕ*(*x*) and a fixed intensity of 0.5 (half of the maximum value shown in Figure 1e) is used. The ‘optimal' planar lens, as another reference case, assumes that all the point sources share an identical intensity and can provide any required *ϕ*(*x*). *f_analytic_* denotes the focal lengths extracted from the focusing patterns obtained from our analytic model. The full-width at half-maximum (FWHM) at *f_analytic_* as a function of the *f_nominal_* is reported in Figure 3a. It can be seen that both the ‘optimal' (black curve) and ‘quasi-optimal' (blue curve) lenses show an increasing FWHM as *f_nominal_* increases, and the FWHM of the ‘quasi-optimal' case is always smaller than the ‘optimal' case. The result for the ‘realistic' planar lens, denoted by the red squares, fluctuates around the two reference curves at *f_nominal_* < 27.5 *µ*m. At large *f_nominal_*, the ‘realistic' and ‘quasi-optimal' planar lenses overlap, because *ϕ*(*x*) is within the phase modulation range at any *x* on the lens plane.

Before commenting on their performances, the focal length error (Δ*f*) defined as |*f_nominal_*-*f_analytic_*| is also reported for the three cases. Δ*f*/*f_nominal_* is plotted in Figure 3b. Note that Δ*f* depends largely on the FN value of a lens^37^ (see Supplementary Section 1 for more details). In contrast to Figure 3a, Δ*f* of the ‘realistic' planar lens does not fluctuate around the two reference curves, for instance, it is larger than both of the references at *f_nominal_* around 10 *µ*m. This observation indeed reflect the effect from the limited phase coverage. Based on the fact that *f_analytic_* of the ‘realistic' planar lens is smaller (larger Δ*f*) than the two references, it is not surprising to see the ‘realistic' planar lens with moderate values of FWHM compared with the two references where *ϕ*(*x*) are completely satisfied for all sources. This is because smaller *f_analytic_* corresponds to a larger effective numerical aperture, and FWHM is inversely proportional to numerical aperture. For the sake of completeness, the phase error (Δ*ϕ*) is shown in Figure 3c. This panel explains the discontinuous behavior of ‘realistic' planar lens in Figure 3a and b. As an example of how Δ*ϕ* is calculated, the case of *f_nominal_* = 7.5 *µ*m is illustrated as an inset in Figure 3c, where the grey bars denote the phase difference at the each source position. As highlighted by the dashed lines across Figure 3a–c, the three plots share the same discontinuous zones for the ‘realistic' planar lens. It can be concluded that the abrupt changes in FWHM and Δ*f* are due to the discontinuities in Δ*ϕ*. The cause of the discontinuities in Δ*ϕ* can be ascribed to the piecewise function which is used to determine the phase and intensity of each source.

At several *f_nominal_* points, the ‘realistic' planar lens does show better performance in terms of both FWHM and Δ*f* compared to the ‘optimal' situation. For instance, in the bands highlighted in blue, the FWHM and Δ*f* of the ‘realistic' planar lens are smaller than those of the ‘optimal' cases. This suggests the phase error Δ*ϕ* as well as the intensity variation does not always interfere with the focusing patterns. Indeed, the process of designing a planar lens can be regarded as a reverse engineering, in which the phase distribution projected from the nominal focal point onto the finite-width lens plane is reconstructed. Because Eq. 2 assumes the focal point radiates electromagnetic field uniformly in every direction and our lens plane is just a few times wider than *λ*, the recorded or reproduced field is only a tiny fraction of the total field radiated from the focal point. In other words, none of the cases considered has abundant information of the focal point due to the small FN value of the lens. Based on these facts, the information introduced by Δ*ϕ* and the slit-to-slit intensity variation, at certain situations, may improve the focusing patterns yielded by our lens as highlighted by the blue band.

**Figure 4:**
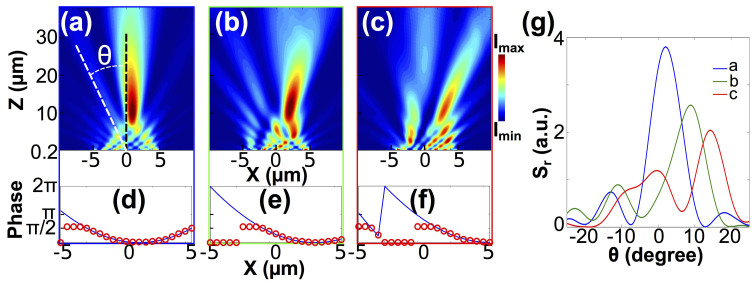
Panels (a), (b) and (c) show the simulated cross-sections of the magnetic intensity distributions of the planar lens with nominal lateral offsets of (a) 1 *µ*m, (b) 3 *µ*m and (c) 5 *µ*m. Nominal focal length is fixed at 20 *µ*m. Panels (d), (e) and (f) show the calculated phase fronts (blue curves) and the discrete phase distributions achieved by arranging the results from the single-slit configuration (red circles) to mimic the calculated curves. (g) Angular-dependent far-field radial component of the time-avaraged Poynting vector *S_r_* as a function of the azimuthal angle *θ*. *θ* is defined as the angle between a line crossing the the center of the lens plane and the lens axis as marked in panel (a).

Once we have shown its ability to engineer the phase front for different *f_nominal_*, we demonstrate next how this platform behaves if the designed focus is off-axis. Three examples of laterally shifted focuses obtained through FDTD simulations are shown in Figure 4a–c, with their corresponding ideal phase fronts and engineered discrete phase distributions shown in Figure 4d–f. Nominal lateral offsets *A_nominal_* are 1, 3 and 5 *µ*m, while *f_nominal_* are set to be 20 *µ*m. The magnetic field intensity clearly evidence the focus deviating away from the lens axis. The coordinates of the focuses in xz-plane are found to be (0.9 *µ*m, 10.3 *µ*m), (2.1 *µ*m, 11.5 *µ*m) and (3.0 *µ*m, 10.8 *µ*m), respectively. The change in the phase front is also corroborated by Figure 4g, which renders the angular-dependent far-field radial component of the time-averaged Poynting vector *S_r_* as a function of the azimuthal angle. It is apparent that a larger *A_nominal_* results in a smaller angular aperture. This means a decreased fraction of information is used to reconstruct the focus, and thus the focusing pattern is expected to degrade at a larger *A_nominal_* value.

**Figure 5:**
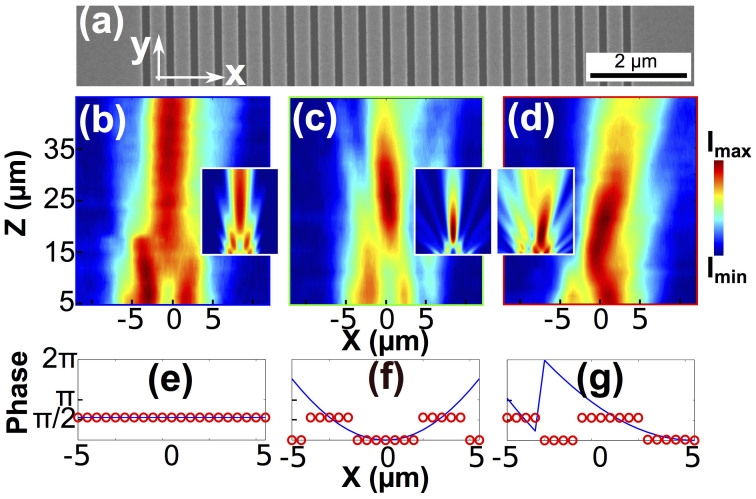
(a) SEM image of the fabricated planar lens before sputtering GST. (b), (c) and (d) Focusing pattern measured in *xz*-plane by confocal scanning optical microscopy for amorphous GST in all slits without crystallization, GST being crystallized in the selected slits in an effect to construct phase fronts similar to Figure 2d and Figure 4f, respectively. Insets of (b), (c) and (d) show the corresponding simulation results of the planar lens in each case using the binarized GST crystallization levels. (e), (f) and (g) The calculated phase fronts *ϕ*(*x*) (blue curves) and the binarized discrete phase distributions (red circles) which are anticipated from the samples in (b), (c) and (d), respectively.

The experimental realization of our planar lens is demonstrated in Figure 5. The structures were fabricated by milling the 21 slits on a 100 nm-thick gold film using focused ion beam (FIB) as shown in Figure 5a. Then 100 nm GST was sputtered on the samples covering the entire surfaces (see Methods Section for more details). To achieve different focus patterns, GST in each slit was supposed to be selectively crystalized to the levels required by Eq. 2 and Figure 1d. However, in our current setup, GST crystallization inside the slits could not be precisely monitored and controlled. In an effort to prove the concept, the GST which was originally designed to be partially crystallized, is now binarized to either amorphous or crystalline phase, depending on whether *ϕ*(*x*) is more or less than half of the maximum phase modulation value (0.56*π*). A 4 mW 532 nm-wavelength CW laser was focused on the samples by a 100× objective and scanned along the slits at a speed of 0.2 *µ*m/s to ensure thoroughly crystalized GST inside the slits. As a proof-of-concept demonstration, we illustrate far-field patterns from three samples, namely a control sample before any GST crystallization, followed by two samples with the focuses either on or off the lens axis achieved by selectively crystallizing the GST.

Confocal scanning optical microscopy was used to characterize the far-field patterns of the structures. A laser beam at 1.55 *µ*m with a spot size much wider than the structures illuminated the samples through the substrate. The transmitted field was probed on the other side of the samples as shown in Figure 5b–d. For each case, the calculated phase front and the anticipated discrete phase distributions from the sample are shown in Figure 5e–g, respectively. The far-field intensity distribution of the lens before GST crystallization is shown in Figure 5b. Since no GST crystallization variation is introduced among the slits, a nearly flat phase front is expected. As can be seen, the depth of focus is long, exceeding the movement range of the stage in z direction. Figure 5c shows the lens with GST symmetrically crystallized about the lens axis, which provides a simplified phase front binarized from the case shown in Figure 2a (*f_nominal_* = 10 *µ*m, *A_nominal_* = 0). Compared with Figure 5b, the entire focus is clearly observed with a significantly reduced focal length and depth of focus. This is expected because of the concave phase front created by the slits with crystallized GST on the top side of the lens. Figure 5d, with GST crystallized in a binary version rounded from Figure 4c (*f_nominal_* = 20 *µ*m, *A_nominal_* = 5 *µ*m), illustrates a clear off-axis focus leaning towards the right-hand side. As a result of the asymmetrical phase front about the lens axis, this figure demonstrates a clear effect of lateral focus tuning compared to Figure 5b and c. The trend of the changes in the measured focusing patterns matches the expectations based on the simulation results, exhibiting that the feasibility and potentiality of our design are encouraging. Further simulation results based on the experimental conditions, namely using only 0% and 90% crystallized GST that correspond to the discrete phase distributions marked by the red circles in Figure 5e–g, are shown in the insets of Figure 5b–d. A relative good agreement is observed between the experimental results and the adapted simulations.

Experimental limitations in our current setup prevented us from achieving real-time manipulating of GST crystallization level to realize dynamical focus tuning on a single device. However, this issue can be overcome by incorporating a design similar to those used in phase-change memories, where GST can be also controlled electrically, with the gold on each side of the slit designed as separated electrodes^24^,^30^. In this way, partial crystallization can be also accessed and monitored by controlling the current between the neighboring electrodes and probing the resistance at the same time. High-precision planarization technique can be employed to remove GST outside of the slits. Together with optimized parameters in GST sputtering process^38^, the quality of the Fabry-Pérot resonance supported by such designs can be further improved.

## Conclusions

In conclusion, we have demonstrated a novel planar lens platform working at 1.55 *µ*m by hybridizing the phase-change material GST inside a metallic slit array. The lens can construct phase front for the transmitted field following different patterns without changing the geometry of the structure. Each of the slits supports a strong FP resonance that can be independently shifted across the spectrum by varying the GST crystallization level. The spectral position of the FP resonance in turn determines the phase of the transmitted field at the monochromatic working wavelength. The lens, which consists of 21 such slits, can be engineered to mimic the theoretical phase front from the equal optical length principle by assigning the designed GST crystallization levels to the corresponding slits. Distinct focusing patterns are demonstrated using the same geometry, and preliminary experimental results prove the feasibility of our ideas. This work provides a promising way to realize tunable micro/nano-scaled integrated photonic components and imaging devices, for which extremely flexible and controllable optical responses are required.

## Methods

### Fabrication and measurements

The gold films of 100 nm thickness were prepared by sputtering at a rate of 1 nm/s on quartz substrates. The slit array was then fabricated by a standard focus-ion beam machine. 100 nm GST was sputtered covering the entire patterned surfaces at a rate of 0.4 nm/s. The GST was crystallized by focusing a 532 nm-wavelength CW laser at a power of 4 mW on the sample surface with a 100× objective, and scanning the laser spot along the slits at a speed of 0.2 *µ*m/s. Although this writing optics is diffraction-limited, it can be applied to our samples because the minimum width of crystallization is 1 *µ*m (two periods of the slits) due to the simplifications we made in the experiment. Confocal scanning optical microscopy from Nanonics was used to characterize the the far-field patterns of the structures. A laser beam at 1.55 *µ*m traveling upwards illuminated the samples from the quartz substrate with a spot size much wider than the samples. On the other side of the sample, a 50 *µ*m-diameter multimode fiber on the top of a 50× objective lens worked as a pinhole to probe the transmitted field. The scanning was realized by a piezoelectric stage moving the sample in xz-plane in steps of 0.1 *µ*m in the *x*-direction and 0.5 *µ*m in the *z*-direction. The photons were collected by an InGaAs IR photon counter over 15 ms integration time. The settings described above offer a good compromise between the scanning area and scanning time. The focusing patterns are resolved because the FWHMs of the focuses are more than 1.5 *µ*m and 10 *µ*m in *x*- and *z*-directions, respectively.

### Simulation

The simulation results shown in Figure 1, 2 and 4 were obtained from Lumerical FDTD, a commercial finite-difference time-domain calculation software. The structures were simulated in 2-dimensional environment surrounded by perfectly matched layers (PML). The plane-wave source was launched from the bottom. The optical properties of amorphous and crystalline GST were obtained from Ref. 31 and the optical properties in the inter-medium phases were calculated using the Lorentz-Lorenz relation (Eq. 1). In Figure 1, a single slit configuration was simulated, with a point monitor fixed at 100 nm above GST-air interface along the central axis of the slit to record the transmitted intensities and phases. In Figure 2 and 4, twenty one parallel slits at a center-to-center separation of 500 nm were simulated. A 2D monitor was used to record the transmitted magnetic field intensities. The corresponding crystallization level was assigned to each GST block to mimic the calculated phase distribution *ϕ*(*x*) based on Eq. 2.

### Analytical modeling

The analytic model based on the planar lens design is built in Matlab. The *xz*-plane is considered. The 21 slits are represented by 21 point sources. The sources are placed along *x*-direction at *x_source_*(*n*) = 0.5 *µ*m × *n* − 5.5 *µ*m, *z* = 0, with *n* being the index of the source. The distance between neighboring sources is 0.5 *µ*m. The sources radiate p-polarized electromagnetic wave into the space. Because the magnetic (H) field is normal to *xz*-plane, we opt to calculate the field using the magnetic component. Each source is predefined with a magnetic intensity 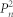 and phase *ϕ_n_*. The effect of surface plasmon excited by the p-polarized light on the gold film is ignored for simplicity. The transmitted field from each slit can be modeled by the Fraunhofer diffraction since *w*^2^/*λ* = 0.015 *µ*m which is much smaller than the distance between the slit and the positions we consider in the space (the slit width *w* = 150 nm and the wavelength *λ* = 1.55 *µ*m). Therefore, the angular distribution of the intensity from each individual source is estimated by the Fraunhofer diffraction pattern, which can be expressed as 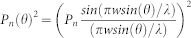, where *w* is the width of the slit (150 nm) and *λ* = 1.55 *µ*m. For a point at coordinate of (x, z) in the 2D plane, the complex H-field contributed by the 21 sources can be express as 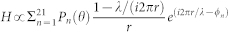, where *θ* = arctan((*x* − *x_source_*(*n*))/*z*) and 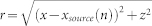. The magnetic field intensity, expressed as |*H*|^2^, is calculated at positions across the area (−10 *µ*m ≤ *x* ≤ 10 *µ*m, 2 *µ*m ≤ *z* ≤ 42 *µ*m) with 0.04 *µ*m and 0.08 *µ*m separations in *x*- and *z*-directions, respective. *f_analytic_* is defined as the *z*-coordinate of the point of the maximum intensity in the focusing region.

## Author Contributions

Y.C. and A.F. designed the structures, X.L. fabricated the samples, Y.C. and Y.S. performed the experiment, Y.C. performed the FDTD simulation and developed the analytic model, X.G.L., M.H. and S.M. co-wrote the manuscript. All authors discussed the results and contributed to the final version of the manuscript.

## Supplementary Material

Supplementary Information Supplementary information
