# Supplementary material for: Engineering the Phase Front of Light with Phase-Change Material Based Planar lenses
Source: Sci Rep. 2015 Mar 2;5:8660. doi: 10.1038/srep08660 (PMC4345347; doi:10.1038/srep08660)
Supplement: Supplementary Information [file srep08660-s1.pdf]

Engineering the Phase Front of Light with Phase-Change Material Based Planar lenses

Yiguo Chen [1,2], Xiong Li[3], Yannick Sonnefraud[4], Antonio I. Fernández-Domínguez[5], Xiangang Luo[3], Minghui Hong[2] and Stefan A. Maier[1*]

[1]*The Blackett Laboratory, Imperial College London, London SW7 2AZ, United Kingdom,*

[2]*Department of electrical and Computer Engineering, National University of Singapore, 117576, Singapore,*

[3]*State Key Laboratory of Optical Technologies on Nano-Fabrication and Micro-Engineering, Institute of Optics and Electronics, Chinese Academy of Science, Chengdu, 610209, China,*

[4]*Inst. NEEL, CNRS, 25 rue des Martyrs BP 166, 38042 Grenoble cedex 9, France,*

[5]*Departamento de Fisica Teorica de la Materia Condensada, Universidad Autonoma de Madrid, E-28049 Madrid, Spain.*

*E-mail: s.maier@imperial.ac.uk

Supplementary Information

Section 1

The comparison of focal length error from lenses with different Fresnel number based on the analytical model

Lens span W is an important parameter when comparing the focusing capability from lenses sharing the same working wavelength λ and nominal focal length $f_{nominal}$. However, since W, λ and $f_{nominal}$ can vary from one design to another, for example, a GHz planar lens with a span of a few centimeters and a nanoscale planar lens working in visible frequencies, a dimensionless gauge is needed to explain the optical responses of the planar lenses across different scales. The Fresnel number (FN), defined as $W^2/(\lambda f_{nominal})$, serves this purpose. A clear study about the effect of FN value on the focal length error is presented in the paper 'On the chromatic aberration of microlenses' (*Opt. Express*, **14**, 4687–4694 (2006)), demonstrating that a lens of small FN has a larger focal length error.

Here, we include a comparison of focal length error between two lenses of different spans (different FN) to show its effect on the lens performance. We use the analytical model of 'optimal' planar lens for the demonstration. The other two analytical models ('quasi-optimal' and 'realistic' planar lenses) are just the derivations of the 'optimal' case by limiting the phase coverage and adding intensity variation.

We report in Supplementary Table S1 on the parameters of the two situations considered. The original design denotes the design we show in the main manuscript with W=10 μm and the expanded design represented the case of W=32 μm. Lens span for the expanded case is chosen in this way so that the ratios among W, λ and $f_{nominal}$ are the same as the design

reported in *Appl. Phys. Lett.* **103,** 183507 (2013). In other words, they have the same FN values, therefore a direct comparison can be established. We keep the spacing between neighboring point sources the same at 0.5 μm, therefore there are 65 point sources in the expanded case. Nominal focal length is set to be 13.95 μm (9λ) for both situations.

	Expanded design	Original design	Ref*
Lens span, W	32 μm (21λ)	10 μm (6.5λ)	111 mm (21λ)
Working wavelength, λ	1.55 μm	1.55 μm	5.29 mm
Point source spacing	0.5 μm	0.5 μm	NA
Number of point sources	65	21	NA
Nominal focal length, $f_{nominal}$	13.95 μm (9λ)	13.95 μm (9λ)	47.62 mm (9λ)
Observed focal length, f	13.67 μm (8.8λ)	8.92 μm (5.8 λ)	49.5 mm (9.35λ)
Focal length error, $\lvert f- f_{nominal} \rvert / f_{nominal}$	2.0%	36.1%	3.9%
The Fresnel number, FN	48.9	4.6	48.9

* Ref. A: Pacheco-Peña, V. *et al.* Ultra-compact planoconcave zoned metallic lens based on the fishnet metamaterial. *Appl. Phys. Lett.* **103,** 183507 (2013).

Supplementary Table S1. The parameters of the two planar lenses

It can be seen that the expanded case has a focal length f at 13.67 μm (8.8λ) and the focal length error is reduced to 2.0% compared to the original desgin. This focal length error is in the same scale as show in the reference paper mentioned above. The FN values are reported in the last row of supplementary Table 1 for comparison.

For the sake of completness, we plot the normalized magnetic intensity distributions along the lens axis for the expanded and original designs, which is shown in Supplementary Fig. 1.

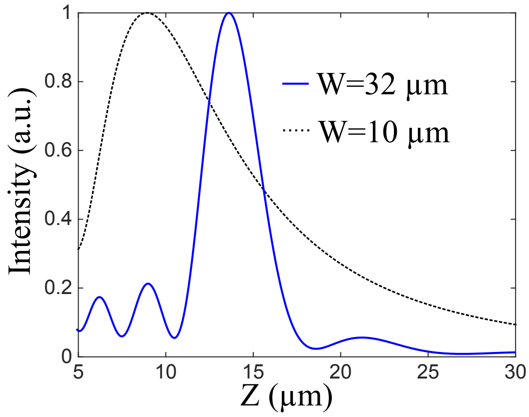

Supplementary Fig. S1. The normalized magnetic intensity distributions along the lens axis

Here we also report the oberseved focal lengths and FWHMs from ‘realistic’ and ‘quasi-optimal’ cases at the expanded W=32 μm. The data are shown in Supplementary Table S2. Clearly, the limitations of the phase coverage and intensities introduce a trade-off between the focal length error and FWHM.

	Optimal	Quasi-optimal	Realistic
Nominal focal length, $f_{nominal}$	13.95 μm (9λ)	13.95 μm (9λ)	13.95 μm (9λ)
Observed focal length, f	13.67 μm (8.82λ)	13.65 μm (8.81 λ)	13.75 μm (8.87λ)
Focal length error, $\|f- f_{nominal}\|/ f_{nominal}$	2.0%	2.2%	1.4%
FWHM	0.81 μm	0.80 μm	0.89 μm

Supplementary Table S2. The comparisons of 'optimal', 'quasi-optimal' and 'realistic' cases at W=32 μm

Section 2

Derivation of Eq. 2

Without Fresnel or Fraunhofer diffraction theory approximations, the 2D integral representation of the scalar electric field diffraction from an aperture is given by

$$E_{diff}(x,z) = E_0 \int_{-W/2}^{W/2} t(x') e^{i\frac{2\pi}{\lambda}\sqrt{(x-x')^2+z^2}} dx',$$

where $t(x')$ is the transmission function, and the integral ranges the whole span of the lens, W. Similarly, the electric field directly transmitted through the aperture reads

$$E_{trans}(x,z) = E_0 e^{i\frac{2\pi}{\lambda}z}.$$

If we choose the position $x_0 = A_{nominal}, f = f_{nominal}$ as the focal point of our lens

$$E_{diff}(x_0,f) = E_0 \int_{-a/2}^{a/2} t(x') e^{i\frac{2\pi}{\lambda}\sqrt{(x_0-x')^2+f^2}} dx',$$

$$E_{trans}(x_0,f) = E_0 e^{i\frac{2\pi}{\lambda}f}.$$

We assume that the transmission of our lens is given by

$$t(x') = \delta(x-x')e^{i\phi(x)}.$$

We have

$$E_{diff}(x_0,f) = E_0 e^{i\left(\frac{2\pi}{\lambda}\sqrt{(x_0-x)^2+f^2}+\phi(x)\right)}.$$

The equal optical path principle states that the phase accumulated by the diffracted waves must be the same as the directly transmitted waves except for a $2\pi n$ factor (where n is an integer), having:

$$\frac{2\pi}{\lambda}\sqrt{(x_0-x)^2+f^2} + \phi(x) = \frac{2\pi}{\lambda}f + 2\pi m.$$

Rearranging

$$\phi(x) = 2\pi m + \frac{2\pi}{\lambda}f - \frac{2\pi}{\lambda}\sqrt{f^2+(x-x_0)^2}.$$

In Fresnel diffraction, the transmitted fields, $z \gg x$ and

$$\sqrt{(x-x')^2+z^2} = z'\sqrt{1+\frac{(x-x')^2}{z^2}} \approx z\left[1+\frac{(x-x')^2}{z^2}\right] = z+\frac{(x-x')^2}{2z}.$$

and

$$E_{diff}(x,z) \approx E_0 e^{i\frac{2\pi}{\lambda}z}\int_{-W/2}^{W/2} t(x')e^{i\frac{\pi}{\lambda z}(x-x')^2}dx'.$$

Using the same expression for $t(x')$ and $E_{trans}(x_0, f)$, we obtain that, under Fresnel approximation, the equal optical path principle leads to

$$\phi(x) = 2\pi m - \frac{\pi}{\lambda f}(x-x_0)^2.$$

In Fraunhofer diffraction, we have

$$\sqrt{(x-x')^2+z^2} \approx z+\frac{(x-x')^2}{2z} = z+\frac{x^2+x'^2-2xx'}{2z} \approx z+\frac{x^2-2xx'}{2z}.$$

where we have assumed that $x'^2 \ll x^2$, $x'^2 \ll 2xx'$ and

$$E_{diff}(x,z) \approx E_0 e^{i\frac{2\pi}{\lambda}z}e^{i\frac{\pi x^2}{\lambda z}}\int_{-W/2}^{W/2} t(x')e^{-i\frac{2\pi}{\lambda z}xx'}dx'.$$

Again, using the same expression for $t(x')$ and $E_{trans}(x_0, f)$, we find that the equal optical path principle under Fraunhofer approximation leads to

$$\phi(x) = 2\pi m - \frac{\pi}{\lambda f}(x_0^2 - 2xx_0).$$

Clearly, the phase distribution described by Eq. 2 in the main manuscript is under neither Fresnel nor Fraunhofer approximations.
